# Lower Levels of Transaminases but Higher Levels of Serum Creatinine in Patients with Acute Hepatitis E in Comparison to Patients with Hepatitis A

**DOI:** 10.3390/pathogens10010060

**Published:** 2021-01-12

**Authors:** Thomas Theo Brehm, Omid Mazaheri, Thomas Horvatits, Marc Lütgehetmann, Julian Schulze zur Wiesch, Ansgar W. Lohse, Susanne Polywka, Sven Pischke

**Affiliations:** 1 Department of Internal Medicine, University Medical Center Hamburg-Eppendorf, 20246 Hamburg, Germany; t.brehm@uke.de (T.T.B.); omid@mazaheri.de (O.M.); t.horvatits@uke.de (T.H.); j.schulze-zur-wiesch@uke.de (J.S.z.W.); a.lohse@uke.de (A.W.L.); 2 German Center for Infection Research (DZIF), Partner Site Hamburg-Lübeck-Borstel-Riems, Germany; mluetgehetmann@uke.de; 3 Institute of Medical Microbiology, Virology and Hygiene, University Medical Center Hamburg-Eppendorf, 20246 Hamburg, Germany; polywka@uke.de

**Keywords:** hepatitis E, HEV, extrahepatic manifestations, renal impairment, serum creatinine, eGFR, hepatitis A, HAV

## Abstract

In patients with hepatitis E virus (HEV) infections, extrahepatic, particularly renal and hematological manifestations, are increasingly reported in the medical literature but have never been studied compared to a control cohort. We retrospectively analyzed medical records of consecutive patients that were diagnosed with acute hepatitis E (AHE) (n = 69) or acute hepatitis A (AHA) (n = 46) at the University Medical Center Hamburg Eppendorf from January 2009 to August 2019 for demographical, clinical, and laboratory information. Patients with AHE had significantly lower median levels of ALAT (798 U/L) and total bilirubin (1.8 mg/dL) compared to patients with AHA (2326 U/L; *p* < 0.001 and 5.2 mg/dL; *p* < 0.001), suggesting a generally less severe hepatitis. In contrast, patients with AHE had significantly higher median serum creatinine levels (0.9 mg/dL vs. 0.8 mg/dL; *p* = 0.002) and lower median estimated glomerular filtration rate (eGFR) (91 mL/min/1.73 m^2^ vs. 109 mL/min/1.73 m^2^; *p* < 0.001) than patients with AHA. Leucocyte, neutrophil and lymphocyte count, hemoglobin, platelets, red cell distribution width (RDW), neutrophil to lymphocyte ratio (NLR), and RDW to lymphocyte ratio (RLR) did not differ between patients with AHE and those with AHA. Our observations indicate that renal but not hematological interference presents an underrecognized extrahepatic feature of AHE, while inflammation of the liver seems to be more severe in AHA.

## 1. Introduction

Hepatitis E virus (HEV) is a single-stranded positive-sense RNA virus and presents one of the most common causative agents of acute hepatitis worldwide [1]. In Europe, infections are predominantly caused by HEV genotype 3, which is usually associated with sporadic self-limited infections and a mainly asymptomatic or mild clinical course [2,3]. Rarely, more severe disease may occur and lead to acute liver failure or acute-on-chronic liver failure in patients with underlying hepatic diseases [4]. Immunocompromised hosts may develop chronic hepatitis E, which can evolve to liver cirrhosis with life-threatening sequelae. In recent years, both acute and chronic HEV infections have increasingly been linked to a broad spectrum of extrahepatic manifestations [5]. While the association of HEV infections and various neurological diseases, particularly neuralgic amyotrophy [6] and Guillain-Barré syndrome [7], has been studied in detail, suspected renal and hematological manifestations still require further investigations. A study from China, where most infections are due to HEV genotypes 1 and 4, recently found that various hematologic laboratory parameters are altered in patients with acute hepatitis E (AHE) compared with healthy controls and demonstrated that red cell distribution width (RDW), neutrophil to lymphocyte ratio (NLR), and RDW to lymphocyte ratio (RLR) are associated with disease severity [8]. However, a systematic study evaluating hematologic laboratory parameters from a region where HEV genotype 3 is endemic is currently still lacking. Evidence suggesting that HEV is associated with renal injury in immunosuppressed patients comes from a study that demonstrated a significant decrease in estimated glomerular filtration rate (GFR) in kidney and liver transplant patients during HEV genotype 3 infections [9]. However, kidney function of solid organ transplant recipients may potentially also be affected by several other factors, e.g., toxicity of calcineurin inhibitors, and data from immunocompetent patients are scarce. Our study aimed to assess the relevance of renal and hematological impairment in a cohort of patients with AHE in a region where HEV genotype 3 is predominant. To achieve this, we systematically compared laboratory parameters indicating renal and hematological manifestations between patients with AHE and a control group of patients with acute hepatitis A (AHA).

## 2. Results

### 2.1. Baseline Characteristics of the Study Population

From January 2009 to August 2019, 165 individuals were diagnosed with HEV infections at our institution. Of those, 26 asymptomatic blood donors without signs of clinically overt hepatitis (ALT < 2 times upper limit of normal), 44 patients with chronic hepatitis E, and 26 immunocompromised patients with AHE were excluded from the study. A total of 69 immunocompetent patients with AHE were subjected to further evaluation. HEV infections were either diagnosed by detection of IgM anti-HEV antibodies by enzyme-linked immunosorbent assay (ELISA) (n = 17) or by positive quantitative or qualitative polymerase chain reaction (PCR) (n = 52). During the same time period, 46 immunocompetent patients were diagnosed with AHA. HAV infections were either diagnosed by detection of IgM anti-HAV antibodies by ELISA alone (n = 21) or in combination with positive quantitative or qualitative PCR (n = 25). In the remaining patients, no PCR was performed. None of the patients in our study cohort received any antiviral treatment. Demographic information and laboratory results are presented in Table 1. The majority of patients with AHE and AHA infections were male, and no statistically significant differences in terms of sex distribution were observed among the groups (male: 65.2% vs. 65.2%; *p* = 1.0). Patients with AHE were significantly older than patients with AHA (median age: 51 years vs. 40 years; *p* = 0.001). The body mass index (BMI) did not differ between the groups (25 kg/m^2^ vs. 25 kg/m^2^; *p* = 0.91). 

### 2.2. Liver Function Tests

**Figure 1:**
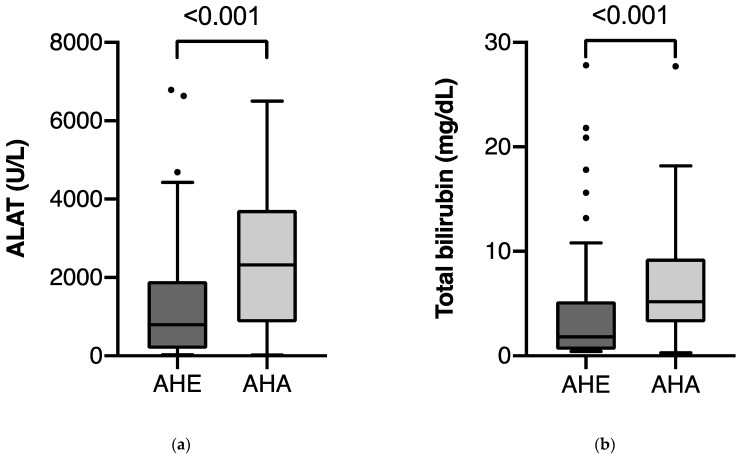

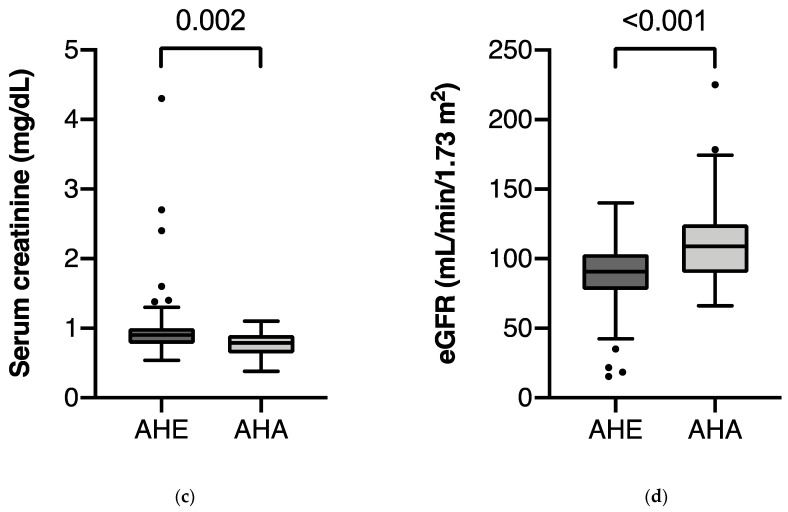
Box-plots of (**a**) alanine aminotransferase (ALAT), (**b**) total bilirubin, (**c**) serum creatinine, and (**d**) eGFR (estimated glomerular filtration rate) of patients with acute hepatitis E (AHE) and acute hepatitis A (AHA). Lower and upper limits of boxes indicate the 25th and 75th percentiles. The horizontal line within boxes indicates the 50th percentile. The lines extending from the boxes indicate the range of nonoutlying values. Outliers are plotted separately as dots.

Compared to hepatitis A patients, patients with AHE presented with significantly lower median levels of alanine aminotransferase (ALAT) (798 U/L vs. 2326 U/L; *p* < 0.001) and median total serum bilirubin (1.8 mg/dL vs. 5.2 mg/dL; *p* < 0.001) than patients with AHA (Table 1, Figure 1a,b).

### 2.3. Hematological Parameters

No significant differences in leucocyte, neutrophil, and lymphocyte count, hemoglobin, platelets, RDW, NLR, and RLR were observed between AHE and AHA patients (Table 1).

### 2.4. Renal Function Parameters

Patients with AHE had significantly higher median serum creatinine levels (0.9 mg/dL vs. 0.8 mg/dL; *p* = 0.002) and significantly lower median estimated glomerular filtration rate (eGFR) (91 mL/min/1.73 m^2^ vs. 109 mL/min/1.73 m^2^; *p* < 0.001) compared to patients with AHA (Table 1, Figure 1c,d). Elevated serum creatinine levels (>1.2 mg/dL) were found in none of the patients with HAV infections and ten (14.5%) patients with AHE. For five of those patients, only the serum creatinine level during the acute phase of HEV infection, but no data prior to infection or after convalescence, were available. The remaining five patients all had higher serum creatinine levels during the acute phase of the HEV than before infection (serum creatinine levels available for three patients) or after convalescence (serum creatinine levels available for five patients) (Supplementary Figure S1). We did not find a correlation between serum creatinine levels and HEV viral load at the time of diagnosis amongst the 52 patients with available HEV viral load at the time of diagnosis (Spearman’s ρ = 0.06; *p* = 0.69). No correlation was detected between serum creatinine levels and age in our study cohort (Spearman’s ρ = 0.08; *p* = 0.42). A multiple regression was run to predict serum creatinine from age and etiology of hepatitis (AHA or AHE). The multiple regression model statistically significantly predicted serum creatinine, *F*(2114) = 4.15, adj. R^2^ = 0.05, *p* = 0.02. Only etiology of hepatitis (*p* = 0.04), but not age (*p* = 0.24) added statistically significantly to the prediction.

## 3. Discussion

The clinical relevance and the pathophysiological link of HEV infections and various assumed extrahepatic manifestations have been under debate in recent years [5]. The present study contributes to this discussion by analyzing a large cohort of patients with AHE from a region where HEV genotype 3 is predominant regarding liver function tests, kidney function tests, and hematologic laboratory parameters compared to a control cohort of AHA patients. Interestingly, we did not observe significant differences in any hematological parameters between patients with AHE and those with AHA. Previous reports have described patients with severely reduced platelet counts during AHE [10,11], suggesting secondary immune thrombocytopenia. Moreover, HEV-RNA has been detected in the bone marrow of patients with acute myeloid leukemia and artificially infected cynomolgus monkeys, which may directly suppress hematopoiesis and thrombopoiesis. However, in our cohort, the overall prevalence of thrombocytopenia was low, and median platelet counts did not differ between patients with AHE and those with AHA. In contrast to a previous investigation from China, where HEV genotypes 1 and 4 predominate [8], RDW, NLR, or RLR in our cohort of HEV infected patients from a genotype 3 region were considerably lower and not significantly different from AHA patients, demonstrating that these parameters are not capable of differentiating between AHA and AHE. The differences between the two studies may be the result of different pathophysiological mechanisms in individuals infected with different HEV genotypes or genetic or immunological host factors. An important finding of our study is that serum creatinine was significantly higher, and eGFR was significantly lower in AHE patients in comparison to AHA infected patients, while laboratory signs of hepatitis were higher in AHA patients. These pilot findings add further evidence to existing research suggesting that HEV infections are associated with renal manifestations. Previous clinical evidence comes from a cohort of solid organ transplant patients, who experienced a significant GFR decline during acute and chronic HEV genotype 3 infections [9]. Five patients in this previously published cohort underwent kidney biopsy, which revealed signs of membranoproliferative glomerulonephritis, IgA nephropathy, or nephroangiosclerosis. Notably, the far majority of HEV infected patients in our study cohort only developed mildly elevated serum creatinine levels, which promptly returned to normal ranges after convalescence. Thus, it can be reasoned that the clinical impact of renal injury should not be overestimated in AHE patients without underlying kidney diseases. Interestingly, these renal findings are despite the fact that HEV patients in our study cohort had a milder hepatitis as reflected by significantly lower ASAT and total bilirubin levels than patients with AHA. These findings suggest that this phenomenon is not an unspecific secondary result of hepatic inflammation but rather reflects an extrahepatic manifestation directly or indirectly attributable to HEV infection. Putative pathophysiological explanations for extrahepatic manifestations of HEV are immune-mediated mechanisms. Viral infections induce a large variety of host defense mechanisms, which are not necessarily restricted to the primary location of infection and may cause systemic or multi-organ disorders. In addition, there is increasing evidence to suggest that HEV also replicates in extrahepatic tissues like the kidneys. Previous studies have detected HEV RNA and antigen in the urine of patients with acute and chronic infections as well as in the kidneys of artificially infected cynomolgus monkeys [12,13]. Viral replication has also been demonstrated in the kidneys of infected rabbits by detection of both positive and negative strands of HEV RNA as well as by immunohistochemical analysis [14]. Besides, HEV has been isolated from neuronal cells [15], placenta [16], the bone marrow [17], and breast milk [18]. These findings challenge the dogma that HEV is an exclusively hepatotropic virus and suggest that renal manifestations of AHE may as well be a result of active extrahepatic viral replication. 

Our study is subject to several limitations. Firstly, due to the retrospective nature of this analysis, the results are limited by the data that were recorded on patient charts. While normal serum creatinine levels prior to infection or after convalescence suggesting transient acute renal dysfunction were available from five patients with AHE, another five patients had no such follow-up serum creatinine levels. Therefore, it cannot be excluded that these individuals suffered from chronic kidney injury. The duration of renal impairment and the kinetics of serum creatinine over time cannot be reliably determined. Secondly, diagnosis of a number of patients with AHA and AHE respectively was established solely by positive serology in combination with clinically overt hepatitis. Since no antibody titers over time were available, it cannot be excluded that some of those serological results presented here are false positive. Thirdly, no kidney biopsies or HEV-PCR from urine samples were performed and only very few data on urine output and urine sediment analyses were available, so we were not able to further characterize the pathophysiology of kidney injury associated with AHE. Lastly, while no HEV genotyping was performed in the present study, it was conducted at a tertiary care hospital in a region where HEV genotype 3 is endemic. Our results may not be generalizable to other healthcare settings or geographical locations where different HEV genotypes predominate. 

## 4. Materials and Methods

Consecutive immunocompetent adult patients diagnosed with AHA or AHE at the University Medical Center Hamburg-Eppendorf between January 2009 and August 2019 were analyzed in this retrospective cohort study. Immunocompromised individuals, patients with chronic hepatitis E, and asymptomatic blood donors with HEV infections without signs of clinically overt hepatitis (ALT < 2 times upper limit of normal) were excluded from the analysis. Diagnosis of HAV infection was established by detecting IgM anti-HAV antibodies by enzyme-linked immunosorbent assay technique (ELISA). HEV infections were either diagnosed by detection of IgM anti-HEV antibodies by ELISA or by positive quantitative or qualitative PCR in combination with clinically overt hepatitis. Demographic, clinical, and laboratory results were obtained by chart review. All laboratory information was evaluated at the day of diagnosis or, if unavailable for this day, between three days before and three days after diagnosis of HAV or HEV infection. The estimated glomerular filtration rate (eGFR) was calculated using MDRD equation [19]. The study was reviewed and approved by the Ethics Committee of the Medical Council of Hamburg (WF-138/20). Continuous variables with a non-normal distribution were expressed as median and interquartile range (IQR) and compared with Mann–Whitney U-test. Categorical variables were expressed as number (%) and compared with Fisher’s exact test. Spearman’s correlation was used to assess the association between serum creatinine and HEV viral load and between serum creatinine and age respectively. A multiple regression was run to predict serum creatinine from age and etiology of hepatitis. P values less than 0.05 were considered statistically significant. Statistical analyses were performed using SPSS, version 21.0 (IBM Corp., Armonk, NY, USA). Figures were designed using GraphPad Prism version 8 for macOS (GraphPad Software, La Jolla, CA, USA).

## 5. Conclusions

In summary, our study indicates that AHE but not AHA may be associated with transient acute renal dysfunction. This phenomenon appears not to be related to the degree of hepatic inflammation, but is likely to either result from a direct viral effect or from immune-mediated mechanisms. In contrast to studies from other geographical locations, hematological manifestations were not commonly observed in this study from a region where HEV genotype 3 is predominant. In the future, prospective multi-center studies are needed to confirm our pilot findings and to unravel the pathophysiological link between AHE and kidney injury.

## Figures and Tables

**Table 1:** Baseline demographics and laboratory parameters of patients with acute hepatitis E and acute hepatitis A.

	AHE	AHA	*p*-Value
n	69	46	
Male—No. (%)	45 (65.2)	30 (65.2)	1.0
Age [years]—Median (IQR)	51 (41–62)	40 (26–51)	0.001
BMI [kg/m^2^]—Median (IQR) ^1^	25 (24–28)	25 (22–30)	0.91
Hemoglobin [g/dL] —Median (IQR)	14.2 (13.4–15.2)	14.0 (13.4–14.9)	0.73
RDW [%]—Median (IQR)	14.4 (13.4–15.2)	13.9 (13.2–15.4)	0.78
Leucocytes [×10^3^/µL]—Median (IQR)	6.0 (4.6–7.3)	5.5 (4.2–6.8)	0.28
Neutrophils [×10^3^/µL]—Median (IQR)	3.4 (2.4–5.2)	3.1 (2.2–3.7)	0.41
Lymphocytes [×10^3^/µL]—Median (IQR)	1.6 (1.0–2.1)	1.8 (1.4–2.3)	0.25
NLR—Median (IQR)	2.3 (1.3–3.5)	1.6 (1.4–2.6)	0.20
RLR—Median (IQR)	8.5 (6.7–15.2)	8.1 (6.1–10.9)	0.29
Platelets [×10^3^/µL]—Median (IQR)	213 (154–264)	247 (192–283)	0.13
ALAT [U/L]—Median (IQR)	798 (185–1912)	2326 (856–3720)	<0.001
Total serum bilirubin [mg/dL]—Median (IQR)	1.8 (0.6–5.2)	5.2 (3.2–9.3)	<0.001
Serum creatinine [mg/dL]—Median (IQR)	0.9 (0.8–1.0)	0.8 (0.6–0.9)	0.002
eGFR [mL/min/1.73 m^2^]—Median (IQR)	91 (78–103)	109 (90–125)	<0.001

^1^ Data on BMI was available for 27 patients with acute hepatitis A (AHA) and 42 patients with acute hepatitis E (AHE). IQR, interquarile range; BMI, body mass index; RDW, red cell distribution width; NLR, neurophil to lymphocyte ratio; RLR, RDW to lymphocyte ratio; ALAT, alanine aminotransferase; eGFR, estimated glomerular filtration rate.

## Data Availability

The data presented in this study are available on request from the corresponding author.

## Supplementary Materials

The following are available online at https://www.mdpi.com/2076-0817/10/1/60/s1, Figure S1: Serum creatinine levels before, during and after the acute phase of HEV infection.

## References

[1] Kamar N., Bendall R., Legrand-Abravanel F., Xia N.S., Ijaz S., Izopet J., Dalton H.R. (2012). Hepatitis E. Lancet.

[2] Guillois Y., Abravanel F., Miura T., Pavio N., Vaillant V., Lhomme S., Le Guyader F.S., Rose N., Le Saux J.C., King L.A. (2016). High proportion of asymptomatic infections in an outbreak of hepatitis E associated with a spit-roasted Piglet, France, 2013. Clin. Infect. Dis..

[3] Izopet J., Tremeaux P., Marion O., Migueres M., Capelli N., Chapuy-Regaud S., Mansuy J.M., Abravanel F., Kamar N., Lhomme S. (2019). Hepatitis E virus infections in Europe. J. Clin. Virol..

[4] Horvatits T., Schulze Zur Wiesch J., Lütgehetmann M., Lohse A.W., Pischke S. (2019). The clinical perspective on hepatitis E. Viruses.

[5] Kamar N., Marion O., Abravanel F., Izopet J., Dalton H.R. (2016). Extrahepatic manifestations of hepatitis E virus. Liver Int..

[6] van Eijk J.J.J., Dalton H.R., Ripellino P., Madden R.G., Jones C., Fritz M., Gobbi C., Melli G., Pasi E., Herrod J. (2017). Clinical phenotype and outcome of hepatitis E virus-associated neuralgic amyotrophy. Neurology.

[7] Stevens O., Claeys K.G., Poesen K., Saegeman V., Van Damme P. (2017). Diagnostic challenges and clinical characteristics of hepatitis E virus-associated guillain-barré syndrome. JAMA Neurol..

[8] Wu J., Zhang X., Liu H., Guo N., Pan Q., Wang Y. (2019). RDW, NLR and RLR in predicting liver failure and prognosis in patients with hepatitis E virus infection. Clin. Biochem..

[9] Kamar N., Weclawiak H., Guilbeau-Frugier C., Legrand-Abravanel F., Cointault O., Ribes D., Esposito L., Cardeau-Desangles I., Guitard J., Sallusto F. (2012). Hepatitis E virus and the kidney in solid-organ transplant patients. Transplantation.

[10] Colson P., Payraudeau E., Leonnet C., De Montigny S., Villeneuve L., Motte A., Tamalet C. (2008). Severe thrombocytopenia associated with acute hepatitis E virus infection. J. Clin. Microbiol..

[11] Fourquet E., Mansuy J.M., Bureau C., Recher C., Vinel J.P., Izopet J., Péron J.M. (2010). Severe thrombocytopenia associated with acute autochthonous hepatitis E. J. Clin. Virol..

[12] Geng Y., Zhao C., Huang W., Harrison T.J., Zhang H., Geng K., Wang Y. (2016). Detection and assessment of infectivity of hepatitis E virus in urine. J. Hepatol..

[13] Bottino F.O., Gardinali N.R., Salvador S.B.S., Figueiredo A.S., Cysne L.B., Francisco J.S., de Oliveira J.M., Machado M.P., Pinto M.A. (2018). Cynomolgus monkeys (Macaca fascicularis) experimentally and naturally infected with hepatitis E virus: The bone marrow as a possible new viral target. PLoS ONE.

[14] Wang L., Xia J., Wang L., Wang Y. (2017). Experimental infection of rabbits with genotype 3 hepatitis E virus produced both chronicity and kidney injury. Gut.

[15] Zhou X., Huang F., Xu L., Lin Z., de Vrij F.M.S., Ayo-Martin A.C., van der Kroeg M., Zhao M., Yin Y., Wang W. (2017). Hepatitis E virus infects neurons and brains. J. Infect. Dis..

[16] Bose P.D., Das B.C., Hazam R.K., Kumar A., Medhi S., Kar P. (2014). Evidence of extrahepatic replication of hepatitis E virus in human placenta. J. Gen. Virol..

[17] Wang L., Yan L., Jiang J., Zhang Y., He Q., Zhuang H., Wang L. (2020). Presence and persistence of hepatitis E virus RNA and proteins in human bone marrow. Emerg. Microbes Infect..

[18] Rivero-Juarez A., Frias M., Rodriguez-Cano D., Cuenca-López F., Rivero A. (2016). Isolation of hepatitis E virus from breast milk during acute infection. Clin. Infect. Dis..

[19] Levey A.S., Bosch J.P., Lewis J.B., Greene T., Rogers N., Roth D. (1999). A more accurate method to estimate glomerular filtration rate from serum creatinine: A new prediction equation. Modification of diet in renal disease study group. Ann. Intern. Med..

