# Supplementary figures and images for: Lower Levels of Transaminases but Higher Levels of Serum Creatinine in Patients with Acute Hepatitis E in Comparison to Patients with Hepatitis A
Source: Pathogens. 2021 Jan 12;10(1):60. doi: 10.3390/pathogens10010060 (PMC7826713; doi:10.3390/pathogens10010060)

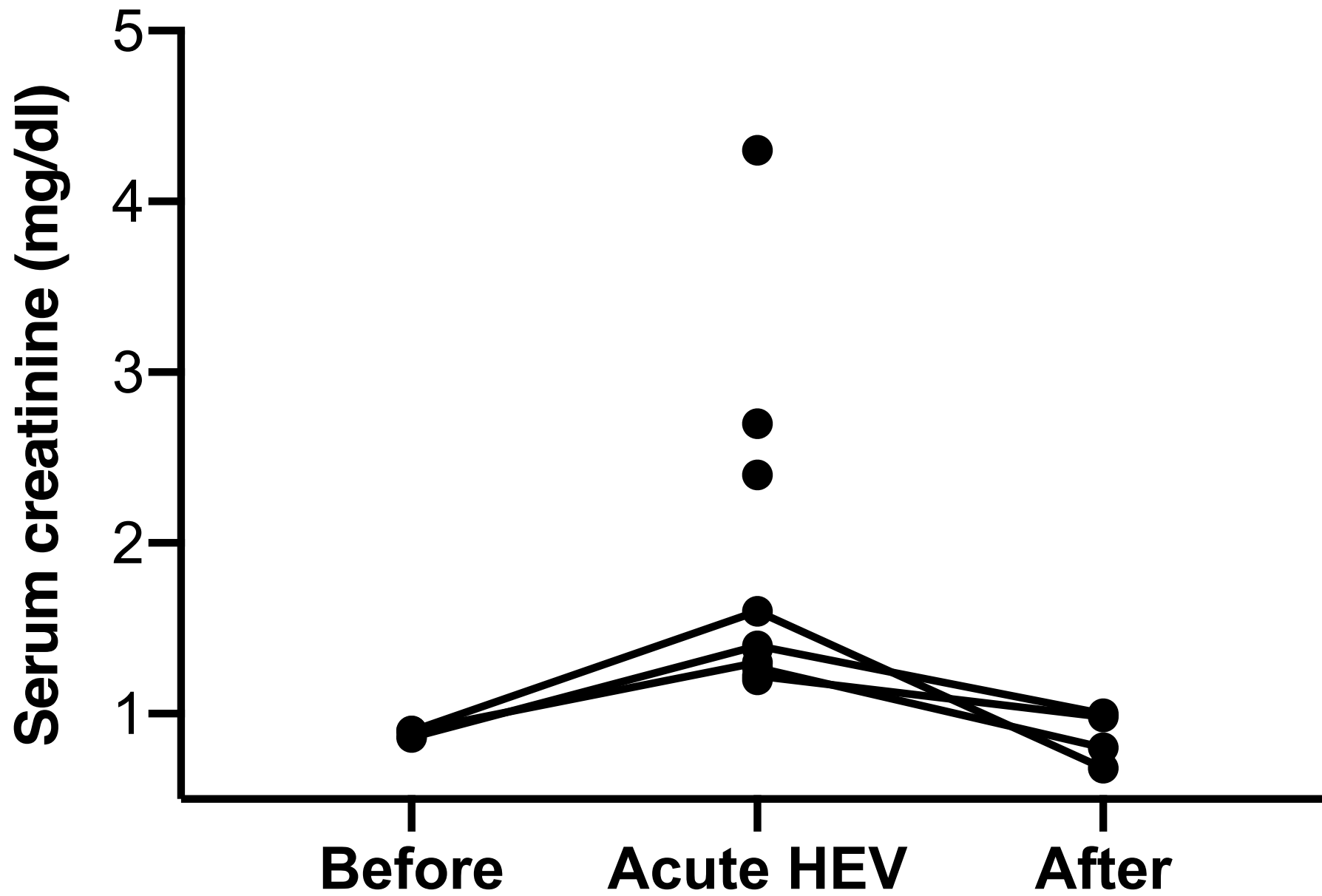

Supplement: Supplementary file 1 [file pathogens-10-00060-s001.zip › Supplementary_table_1.pdf]
